# Supplementary material for: CRISPR targeting of H3K4me3 activates gene expression and unlocks centromere-proximal crossover recombination in Arabidopsis
Source: Nat Commun. 2025 Oct 31;16:9587. doi: 10.1038/s41467-025-65167-3 (PMC12578922; doi:10.1038/s41467-025-65167-3)
Supplement: Supplementary file 1 — Supplementary Information [file 41467_2025_65167_MOESM1_ESM.pdf]

SUPPLEMENTARY INFORMATION:

CRISPR targeting of H3K4me3 activates gene expression and unlocks centromere-proximal crossover recombination in Arabidopsis

Jenia Binenbaum[1,*], Vanda Adamkova[1,*], Hannah Fryer[1], Linhao Xu[1], Nicola Gorringe[1], Piotr Włodzimierz[1,2], Robin Burns[1], Ashot Papikian[3,4], Steven E. Jacobsen[3,5], Ian R. Henderson[1], C. Jake Harris[1,#]

[1] Department of Plant Sciences, University of Cambridge, Cambridge CB2 3EA, UK.
[2] Current Address: Institute of Biochemistry and Biophysics, Polish Academy of Sciences, Warsaw, Poland.
[3] Department of Molecular, Cell and Developmental Biology, University of California, Los Angeles, CA, USA.
[4] Current Address: Plant Molecular and Cellular Biology Laboratory, Salk Institute for Biological Studies, La Jolla, CA 92037, USA.
[5] Howard Hughes Medical Institute, University of California, Los Angeles, CA, USA.
* These authors contributed equally.
[#] For correspondence: cjh92@cam.ac.uk

SUPPLEMENTARY FIGURE LEGENDS:

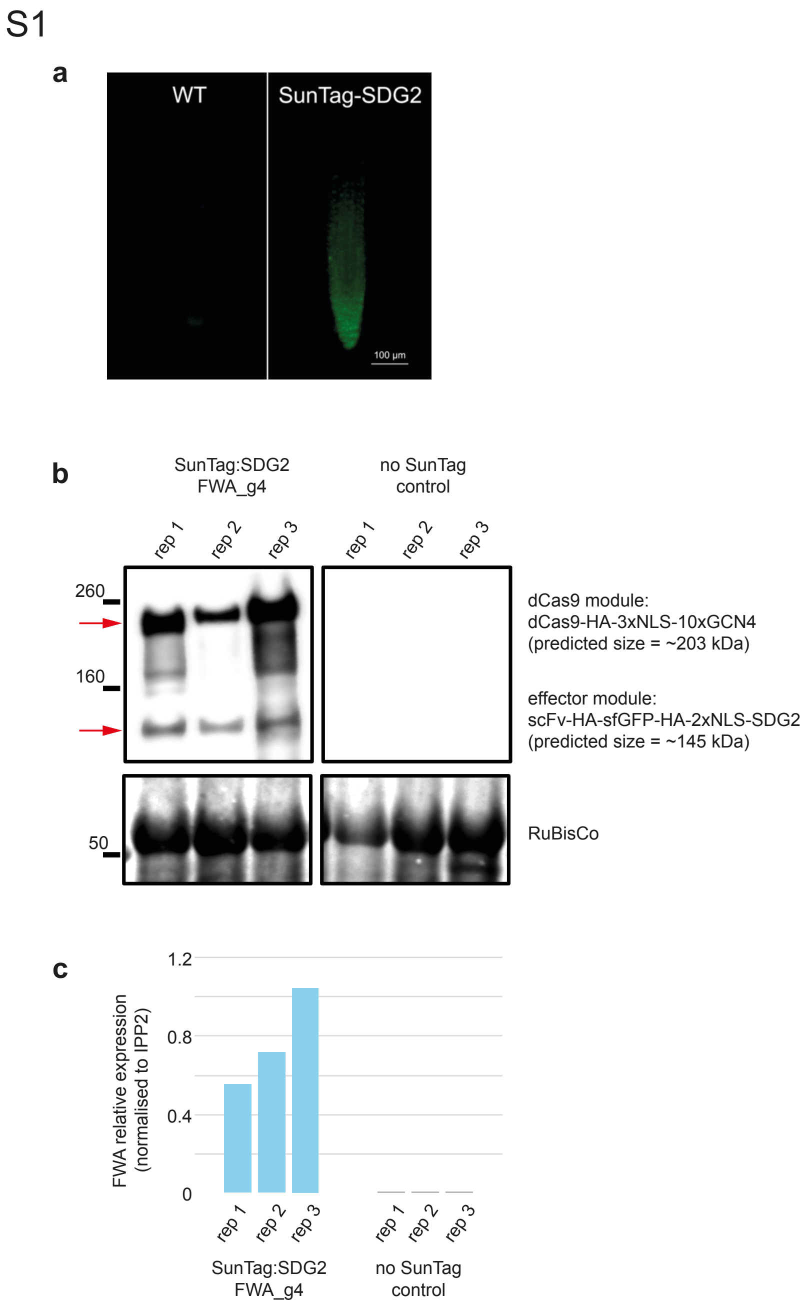

Supplementary Figure 1: A) epifluorescence image of rootstocks from 2-week old representative plants showing nuclear localisation of sfGFP from SunTag lines. B) Western blot for the dCas9-10xGCN4 and effector module in SunTag lines showing activation of *FWA* expression by RT-qPCR show in C). RT-qPCR for *FWA* relative expression (normalised to *IPP2*) from the same individual plants as used for western blot, shown in B).

S2

a SDG2 alignment over SET domain:

```
SDG2   1858 ANYASRICHSCRPNCEAKVTAVDG----HYQIGIYSVRAIEYGEEITFDYNSVTESKEEY 1913
SUVH4   544 GNFARFINHSCEPNLFVQCVLSSHQDIRLARVVLFAADNISPMQELTYDYGYALDSVHGP  603
CLF     821 GDKLKFANHSPEPNCYAKVIMVAG----DHRVGIFAKERILAGEELFYDYRYEPDRAPAW  876
```

b CONSURF analysis:

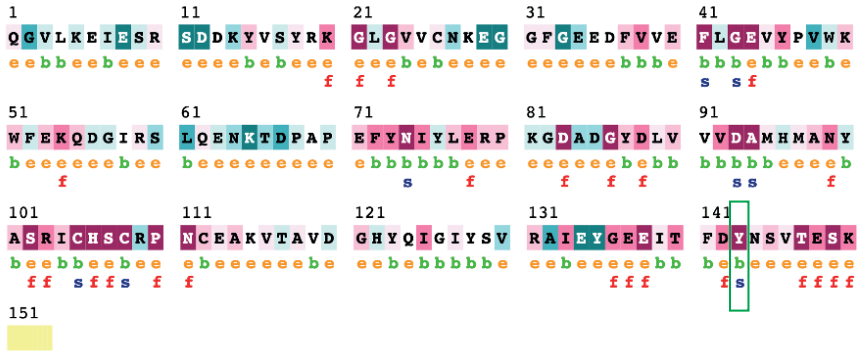

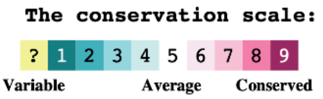

e - An exposed residue according to the neural network algorithm.

b - A buried residue according to the neural network algorithm.

f - A predicted functional residue (highly conserved and exposed).

s - A predicted structural residue (highly conserved and buried).

Supplementary Figure 2: A) Amino acid alignment between SDG2 and two other well characterised A. thaliana histone methyltransferases, SUVH4 (H3K9me2) and CLF (H3K27me3). The Y at amino acid position 1903 of SDG2 that was mutated to F in the SunTag:dSDG2 constructs corresponds to the Y593F in SUVH4(/KYP) that has previously been shown to abolish methyltransferase catalytic activity (Du et al., 2014) (position indicated in green rectangle). B) CONSURF analysis (Ashkenazy et al., 2016) on the SDG2 predicted SET domain (amino acids 1761-1911) showing the maximum level of conservation for SDG2$_{Y1903}$ (position indicated in green rectangle).

S3

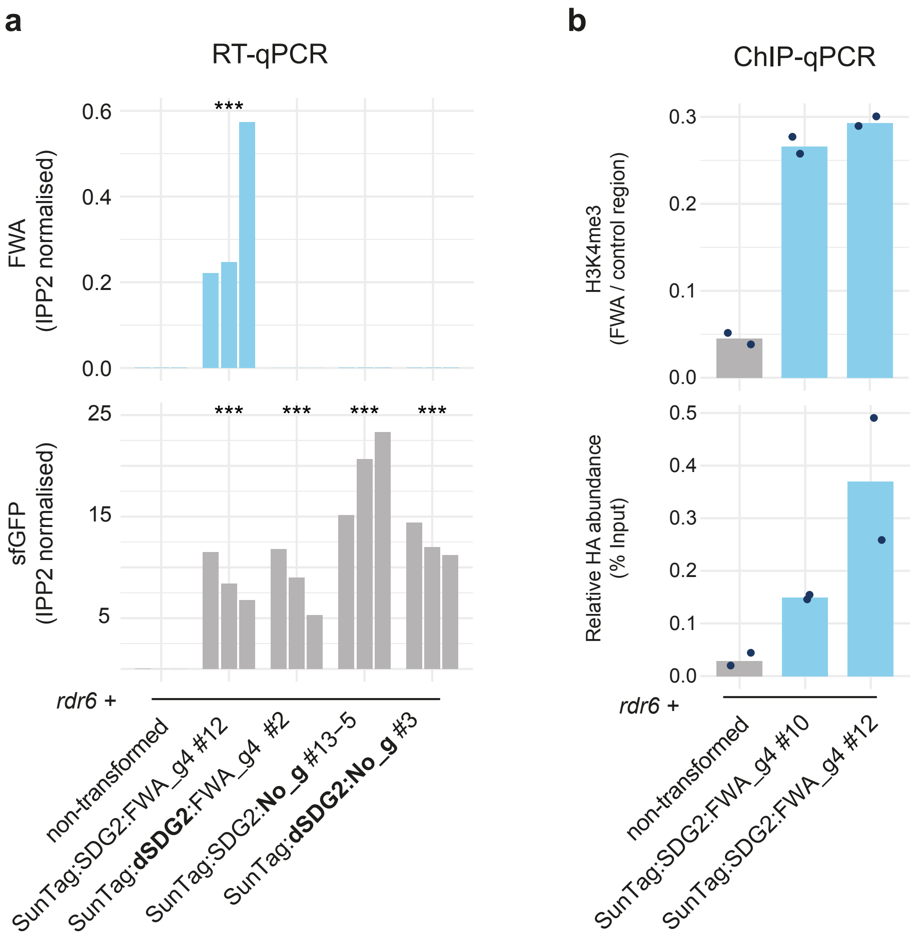

Supplementary Figure 3: A) qRT-PCR for the expression of FWA (upper panel) and sfGFP (lower panel) with individual biological replicates shown. *** indicates significant difference by ANOVA with post-hoc Tukey HSD for FWA, and Dunnett's for sfGFP (with non-transformed being control group), $p < 0.05$. B) ChIP qPCR with two independent FWA targeting lines.

S4

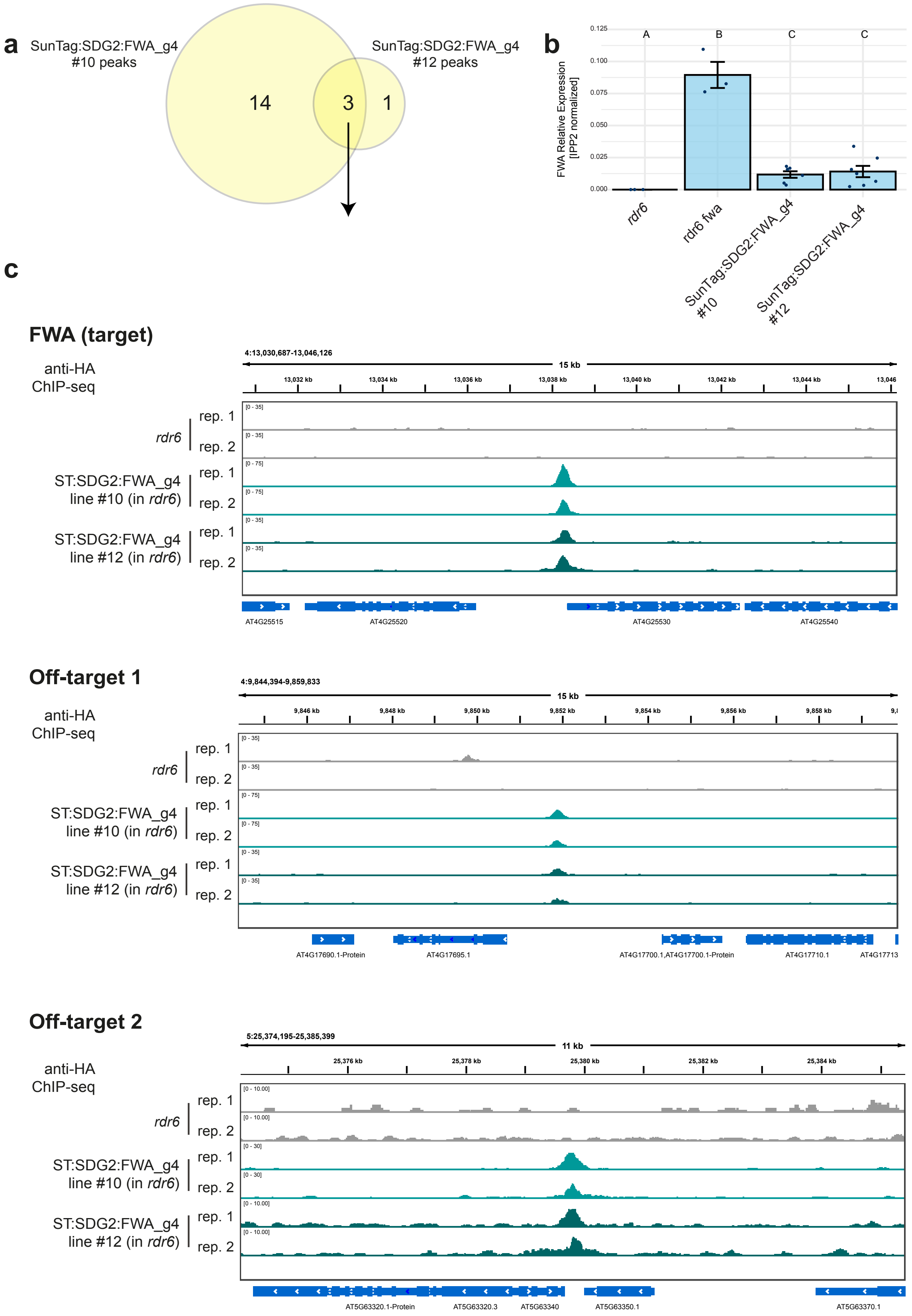
a
SunTag:SDG2:FWA_g4
#10 peaks
SunTag:SDG2:FWA_g4
#12 peaks
14
3
1
b
FWA Relative Expression
[IPP2 normalized]
0.125
0.100
0.075
0.050
0.025
0.000
A
B
C
C
rdr6
rdr6 fwa
SunTag:SDG2:FWA_g4
#10
SunTag:SDG2:FWA_g4
#12
c
FWA (target)
anti-HA
ChIP-seq
rdr6
rep. 1
rep. 2
ST:SDG2:FWA_g4
line #10 (in rdr6)
rep. 1
rep. 2
ST:SDG2:FWA_g4
line #12 (in rdr6)
rep. 1
rep. 2
4:13,030,687-13,046,126
15 kb
AT4G25515
AT4G25520
AT4G25530
AT4G25540
Off-target 1
anti-HA
ChIP-seq
rdr6
rep. 1
rep. 2
ST:SDG2:FWA_g4
line #10 (in rdr6)
rep. 1
rep. 2
ST:SDG2:FWA_g4
line #12 (in rdr6)
rep. 1
rep. 2
4:9,844,394-9,859,833
15 kb
AT4G17690.1-Protein
AT4G17695.1
AT4G17700.1,AT4G17700.1-Protein
AT4G17710.1
AT4G17713
Off-target 2
anti-HA
ChIP-seq
rdr6
rep. 1
rep. 2
ST:SDG2:FWA_g4
line #10 (in rdr6)
rep. 1
rep. 2
ST:SDG2:FWA_g4
line #12 (in rdr6)
rep. 1
rep. 2
5:25,374,195-25,385,399
11 kb
AT5G63320.1-Protein
AT5G63320.3
AT5G63340
AT5G63350.1
AT5G63370.1

Supplementary Figure 4: A) Venn diagram showing the overlap between the peaks called from HA ChIP-seq of two independent SunTag:SDG2 *FWA* targeting lines. B) RT-qPCR analysis of *FWA* expression in two independent SunTag:SDG2:FWA_g4 lines. Dots represent individual biological replicates (a single plant) from the lines (#) and genotypes indicated. Error bars represent SEM. Different letters indicate significant difference by ANOVA with post-hoc Tukey HSD (p < 0.05). n = 3 for *rdr6* and *rdr6 fwa,* n = 6 for SunTag:SDG2:FWA_g4 #10 and n = 7 for line #12. Note that while line #10 appears to bind more strongly to the *FWA* target than line #12, this does not correspond to a significant difference in *FWA* activation. C) Genome browser images for the overlapping peak regions, the top most significant peak is FWA, while the other two are off-target regions with sequence similarity to the FWA binding site that were previously identified (Papikian et al., 2019).

S5

a

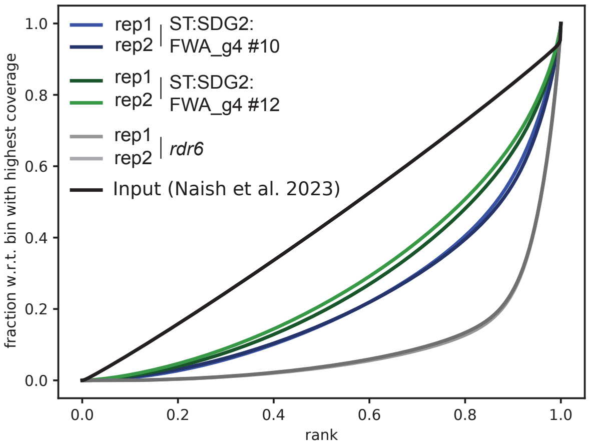

b

Sample	Total mapped reads	mapped reads in peaks	FrIP (%)
SunTag:SDG2:FWA_g4 #10 rep 1	16218459	7845584	48.4
SunTag:SDG2:FWA_g4 #10 rep 2	20650830	10570918	51.2
SunTag:SDG2:FWA_g4 #12 rep 1	12360421	4619230	37.4
SunTag:SDG2:FWA_g4 #12 rep 2	18237850	7013976	38.5
rdr6 rep1	20389796	16361705	80.2
rdr6 rep2	18015360	14238366	79.0

Supplementary Figure 5: Quality control for H3K4me3 ChIPseq of FWA targeting SunTag:SDG2 lines. A) Fingerprint plot (DeepTools) B) Table of total mapped reads and fragments of reads in peaks (FRiP) scores.

S6

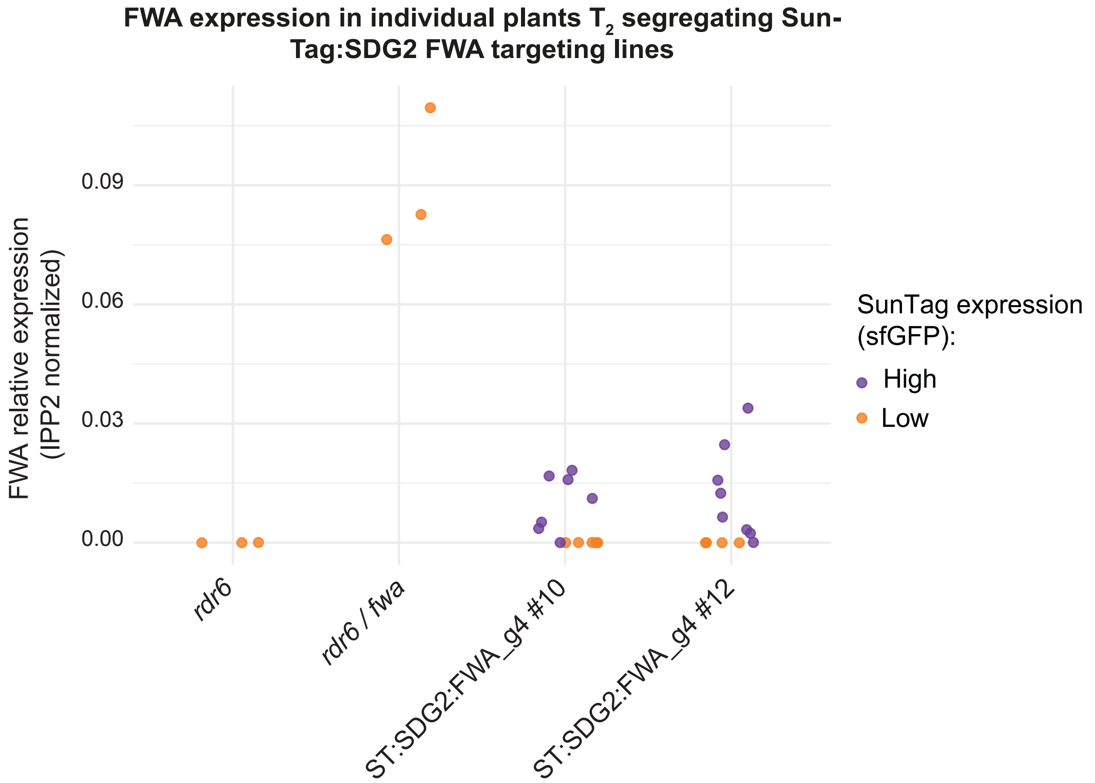

Supplementary Figure 6: Transgenerational stability of *FWA* activation. Plot depicting level of *FWA* expression (RT-qPCR) in T_2 plants from two independent lines segregating for the SunTag:SDG2:FWA_g4 (+/- indicated in purple/orange, respectively). Each dot represents a single T_2 plant. n = 3 for *rdr6* and *rdr6 fwa*, n = 12 for *SunTag:SDG2:FWA_g4 #10* and *#12*.

S7

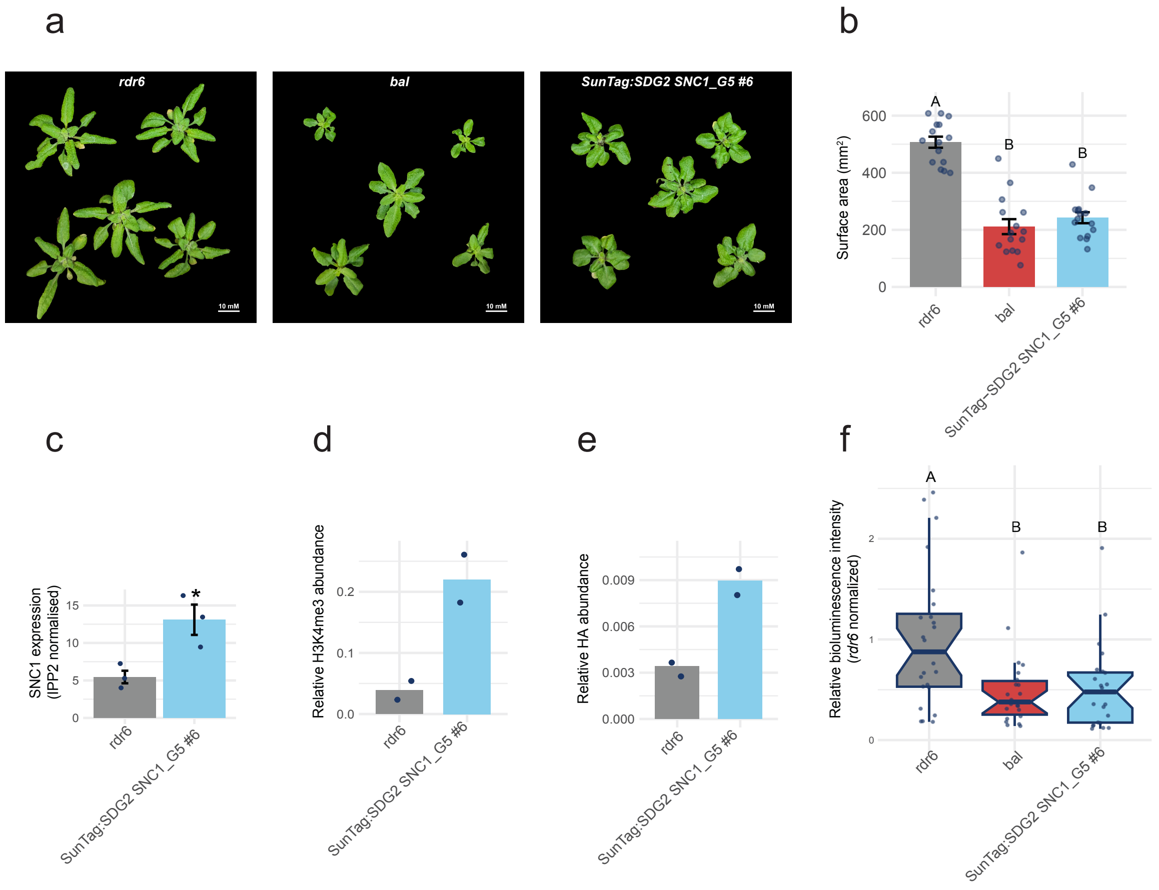

Supplementary Figure 7: Independent SunTag:SDG2:SNC1_g5 T_3 line targeted to *SNC1* enhances resistance to *P. syringae*. A) representative images of 3-week old plants from the genotypes indicated. Scale bar indicates 1cm. B) Rosette surface area quantification. Each dot represents an individual plant. Error bars represent SEM Different letters indicate significant difference by ANOVA with post-hoc Tukey HSD ($p < 0.05$). n = 15. C) RT-qPCR for *SNC1* expression. Error bars represent SEM. * indicates p-value < 0.05 evaluated by Student's T-Test. n = 3. D) ChIP qPCR for H3K4me3 at *SNC1*. E) ChIP qPCR for the presence of SunTag at *SNC1*. F) *Pst::LUX* assay for colonisation quantification at 3 days post inoculation. Different letters indicate significant difference by ANOVA with post-hoc Tukey HSD ($p < 0.05$). n = 24.

S8

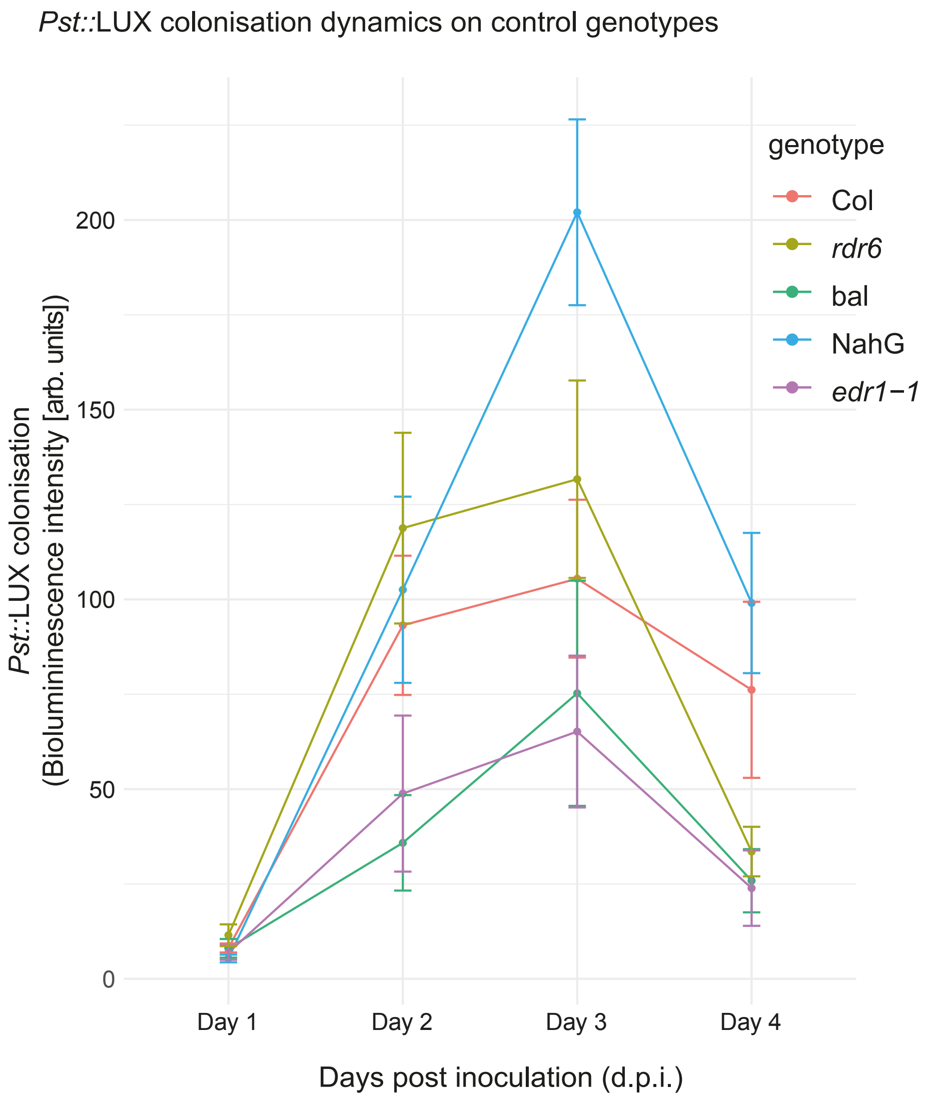

Supplementary Figure 8: Colonisation dynamics of *Pst::LUX* in the genotypes indicated. Error bars represent SEM. n = 8 for Col-0, n = 18 for *rdr6*, n = 10 for *bal*, n = 11 for *NahG* and n = 7 for *edr1-1*.

S9

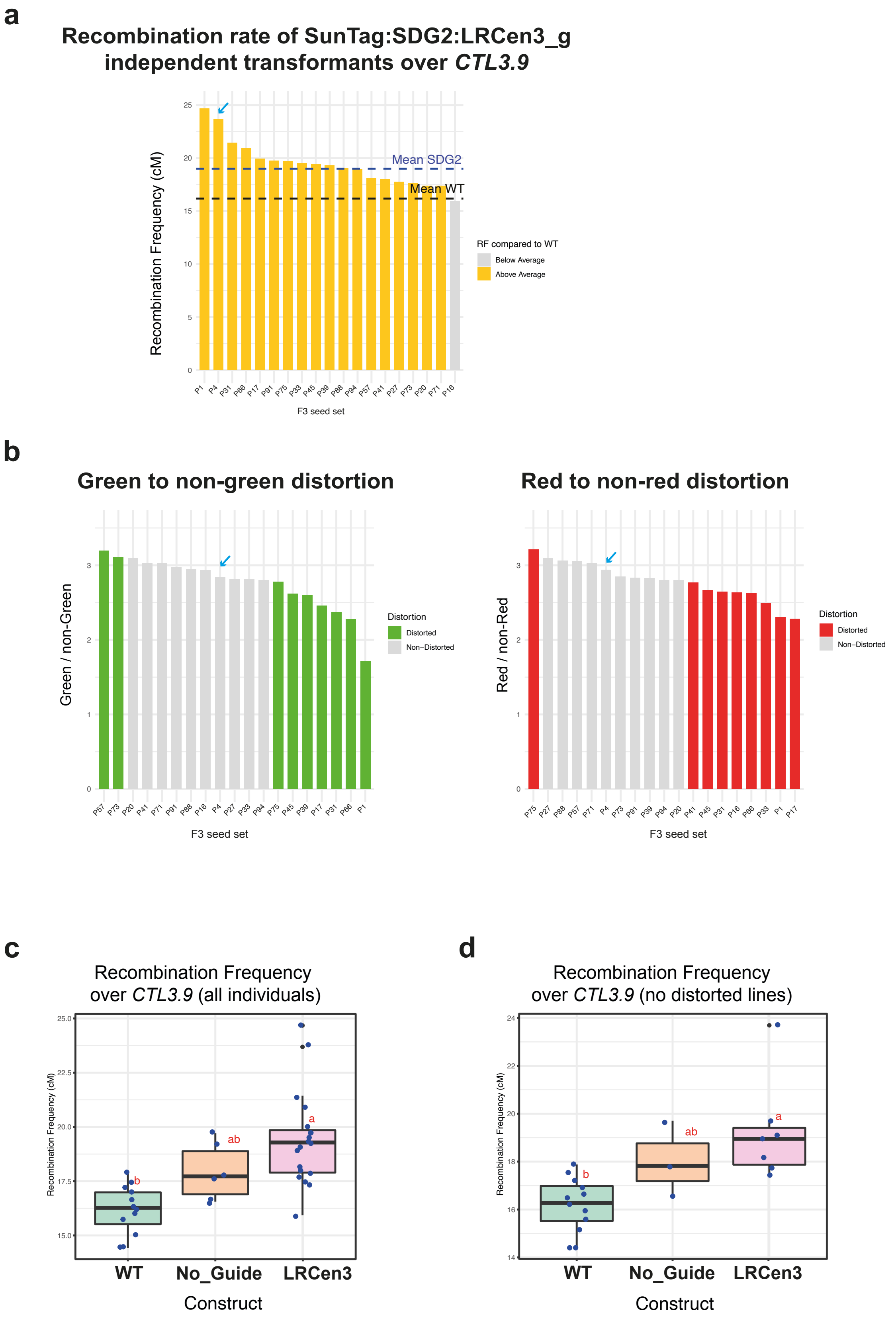
a
Recombination rate of SunTag:SDG2:LRCen3_g
independent transformants over CTL3.9
Recombination Frequency (cM)
Mean SDG2
Mean WT
RF compared to WT
Below Average
Above Average
P1 P4 P31 P66 P17 P91 P75 P33 P45 P39 P88 P94 P57 P41 P27 P73 P20 P71 P16
F3 seed set
b
Green to non-green distortion
Green / non-Green
Distortion
Distorted
Non–Distorted
P57 P73 P20 P41 P71 P91 P88 P16 P4 P27 P33 P94 P75 P45 P39 P17 P31 P66 P1
F3 seed set
Red to non-red distortion
Red / non-Red
Distortion
Distorted
Non–Distorted
P75 P27 P88 P57 P71 P4 P73 P91 P39 P94 P20 P41 P45 P31 P16 P66 P33 P1 P17
F3 seed set
c
Recombination Frequency
over CTL3.9 (all individuals)
Recombination Frequency (cM)
WT
No_Guide
LRCen3
Construct
d
Recombination Frequency
over CTL3.9 (no distorted lines)
Recombination Frequency (cM)
WT
No_Guide
LRCen3
Construct

Supplementary Figure 9: A) Recombination rate over *CTL3.9* of the F_3 lines (as shown in main Figure 3). Blue arrow (also shown in B) points the P4 line seed set taken to the next generation (also see main Figure 3C, red dot). B) Distortion ratios for the green to non-green (left) and red to non-red (right) T-DNAs from *CTL3.9* from the F_3's. C) Recombination rate over *CTL3.9* (F_3's) including all scorable individuals including no guide lines. n = 12 for control, n = 19 for ST:SDG2-LRCen3 and n = 6 for ST:SDG2-NoGuide. D) Recombination rate over *CTL3.9* (F_3's) filtered for individuals with no distortion (i.e. 3:1). n =12 for control, n = 7 for ST:SDG2-LRCen3 and n = 3 for ST:SDG2-NoGuide. For C) and D) different letters indicate significant difference by ANOVA with post-hoc Tukey HSD ($p<0.05$).

S10

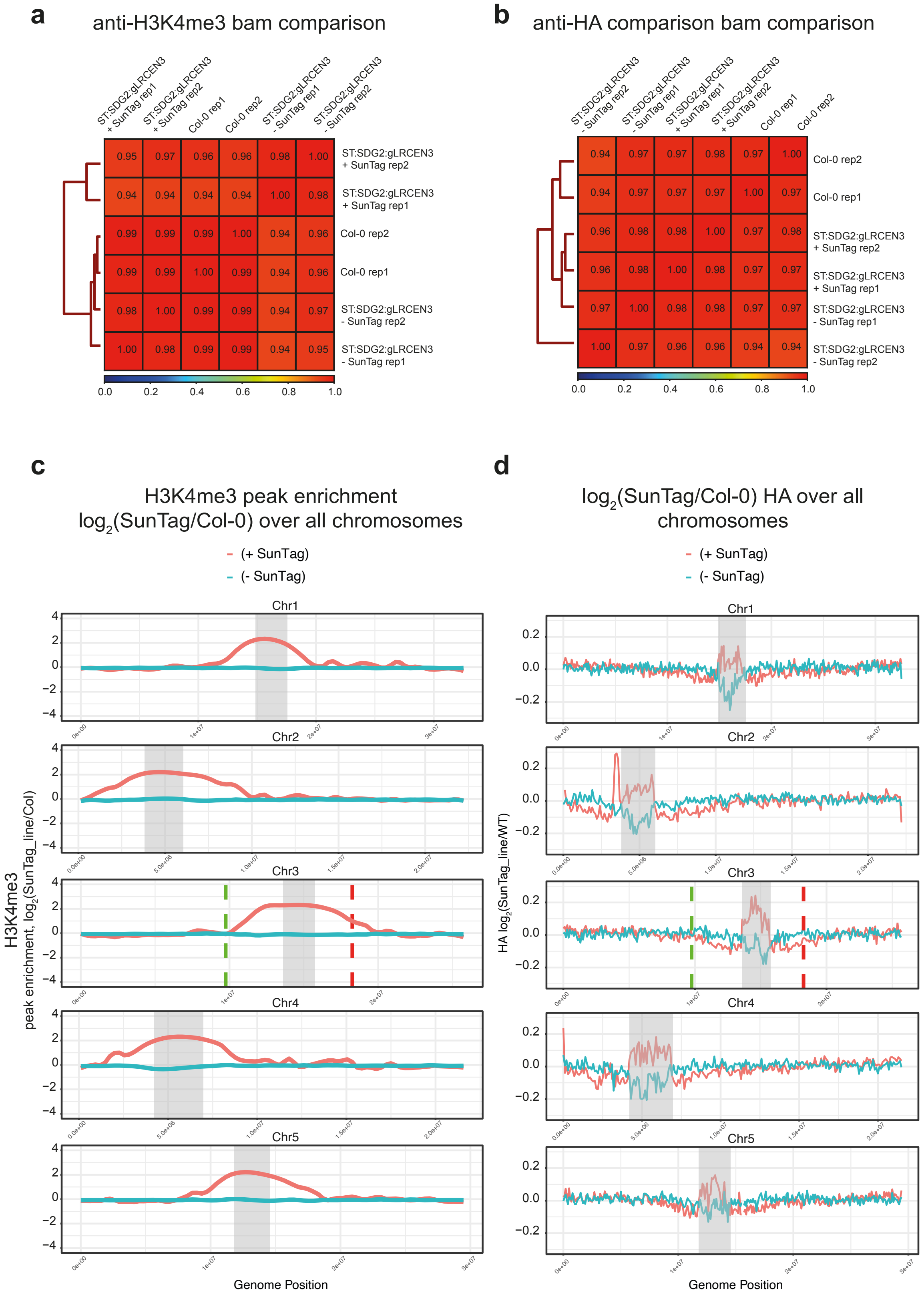
a
anti-H3K4me3 bam comparison
ST:SDG2:gLRCEN3 + SunTag rep1
ST:SDG2:gLRCEN3 + SunTag rep2
Col-0 rep1
Col-0 rep2
ST:SDG2:gLRCEN3 - SunTag rep1
ST:SDG2:gLRCEN3 - SunTag rep2
b
anti-HA comparison bam comparison
c
H3K4me3 peak enrichment
log2(SunTag/Col-0) over all chromosomes
(+ SunTag)
(- SunTag)
Chr1
Chr2
Chr3
Chr4
Chr5
H3K4me3
peak enrichment, log2(SunTag_line/Col)
Genome Position
d
log2(SunTag/Col-0) HA over all chromosomes
HA log2(SunTag_line/WT)

Supplementary Figure 10: A-B) ChIP-seq replicate comparisons by Pearsons correlation clustering using 25bp bin resolution. C-D) ChIP-seq data chromosomal-wide plots as shown in Figure 3, over all chromosomes from sibling lines with or without SunTag:SDG2:LRCen3 (+/-). Enrichment is calculated as log_2 fold change over non-transgenic (Col-0) controls in 100kb windows. *CTL3.9* marker positions are indicated, and centromeric regions are shown in grey.

S11

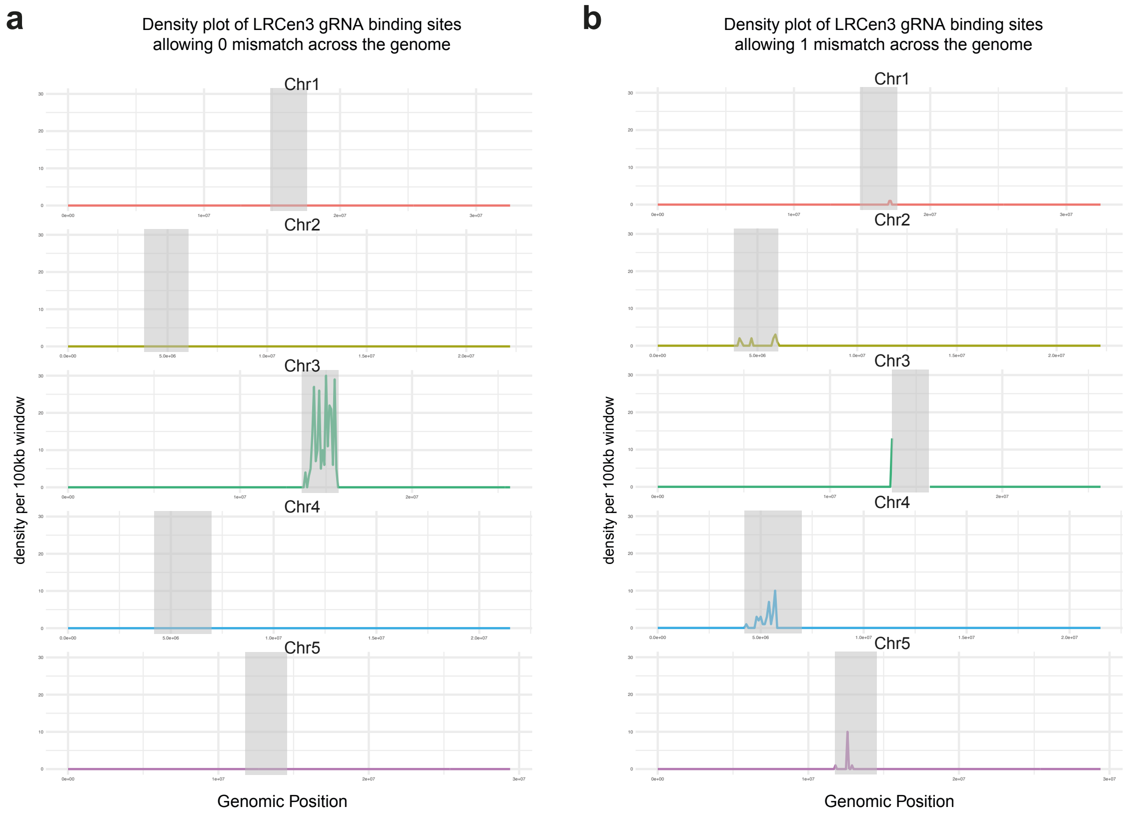

Supplementary Figure 11: A) LRCen3 guide RNA binding site density plot with 0 mismatches or B) with 1 mismatch tolerance.

S12

Centromeric H3K4me3 peaks in SunTag:SDG2:LRCen3 P4 descendents (Col-CEN assembly)

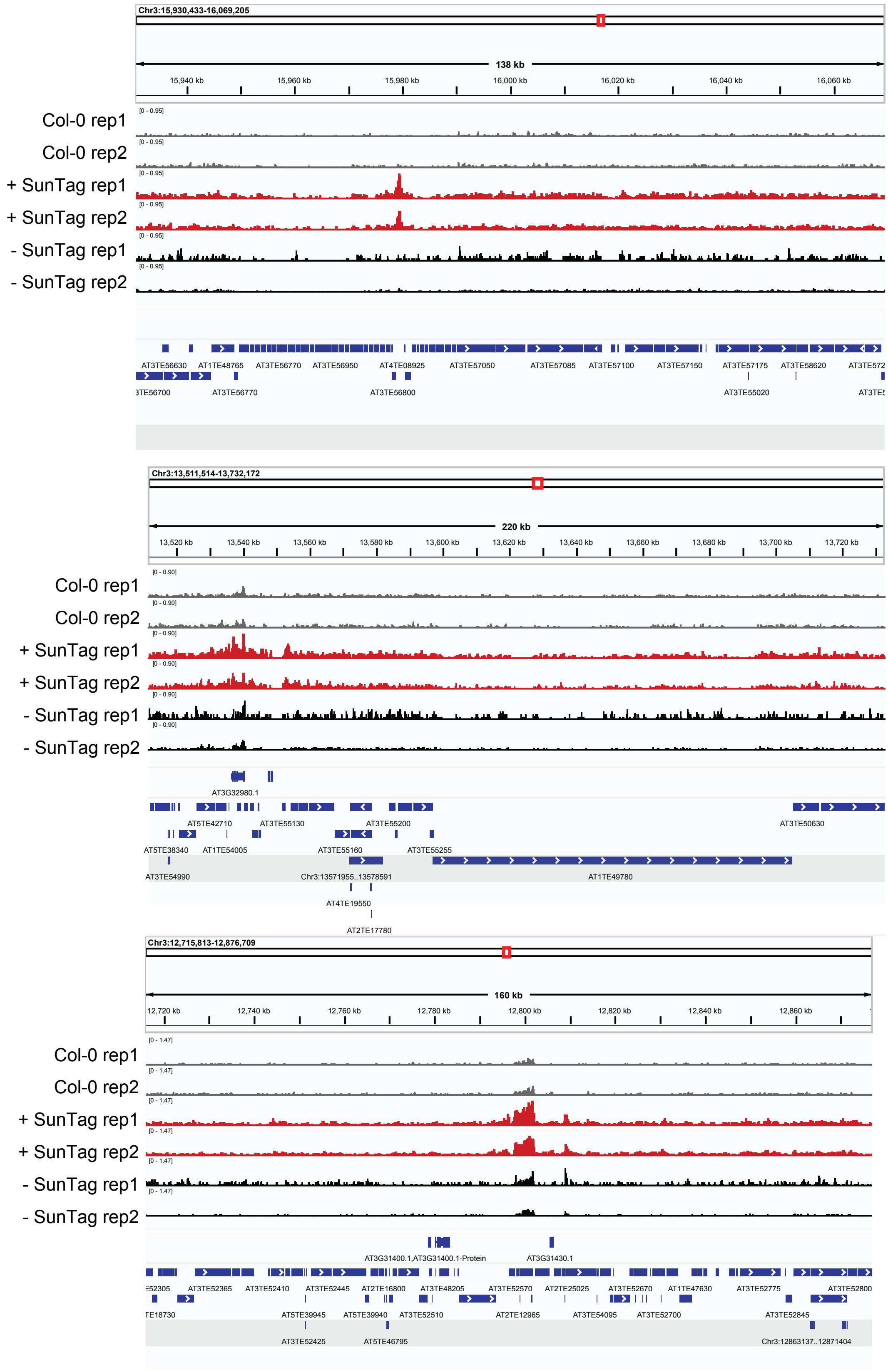

Supplementary Figure 12: Representative genome browser images showing examples of centromeric regions where the SunTag:SDG2:LRCen3_g lines show novel or enriched H3K4me3 peaks.

S13

a

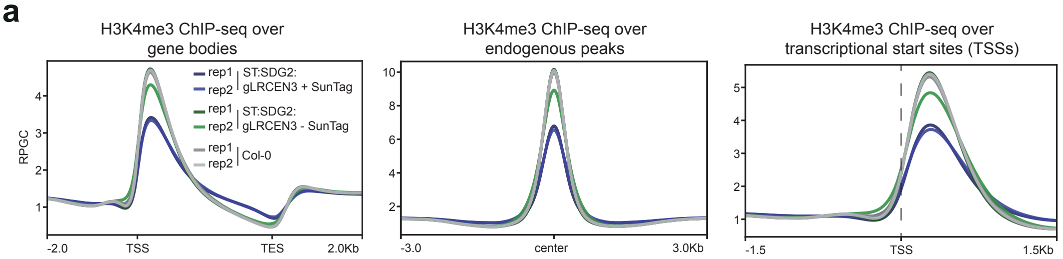

b

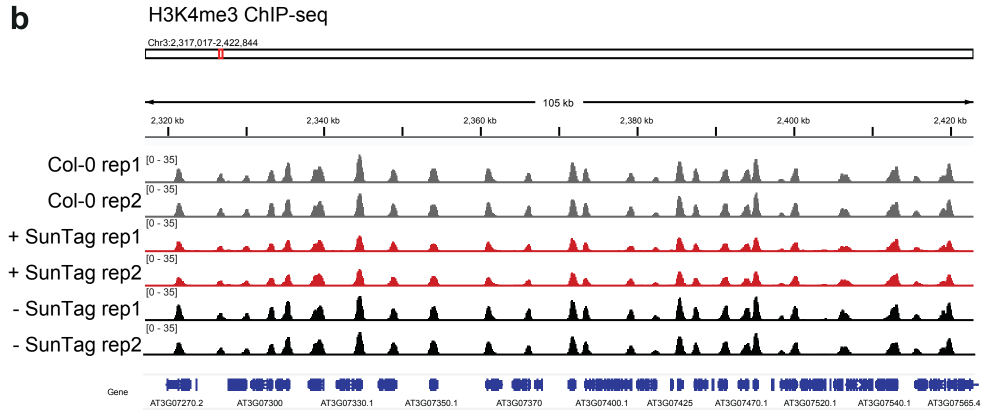

c

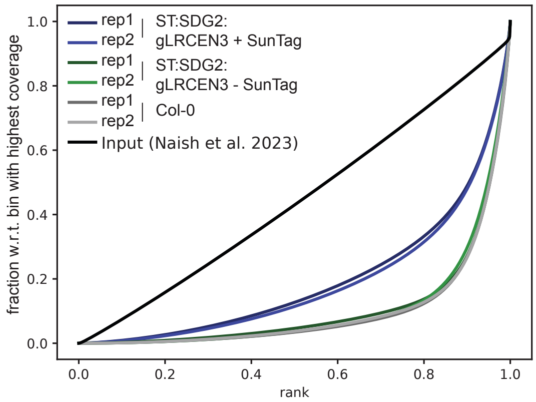

d

Sample	Total mapped reads	mapped reads in peaks	FrIP (%)
anti-H3K4me3 ST:SDG2:gLRCEN3_withST rep1	37124918	22335336	60.2
anti-H3K4me3 ST:SDG2:gLRCEN3_withST rep2	46863315	28851483	61.6
anti-H3K4me3 ST:SDG2:gLRCEN3_noST rep1	50026065	40566813	81.1
anti-H3K4me3 ST:SDG2:gLRCEN3_noST rep2	43236790	34269474	79.3
anti-H3K4me3 Col0 rep1	44335789	36150112	81.5
anti-H3K4me3 Col0 rep2	34744674	28141433	81.0

Supplementary Figure 13: Quality control for H3K4me3 ChIPseq of Cen3 targeting SunTag:SDG2 lines. A) Metaplots over genes, H3K4me3 endogenous peaks, TSS regions in the genotypes indicated B) Representative browser image showing the quality of H3K4me3 ChIP-seq data in the genotypes indicated. C) Fingerprint plot (DeepTools) D) Table of total mapped reads and fragments of reads in peaks (FRiP) scores. Note these are mapped to the TAIR10 assembly (instead of Col-CEN as in Fig. 3 and Supp. Fig. 10-12) to ensure consistency of annotations and for comparison with data shown data from *FWA* targeting SDG2 and PRDM9 SunTag lines in Fig. 1E and Fig. 4E, Supp. Fig. 5 and Supp. Fig. 20.

S14

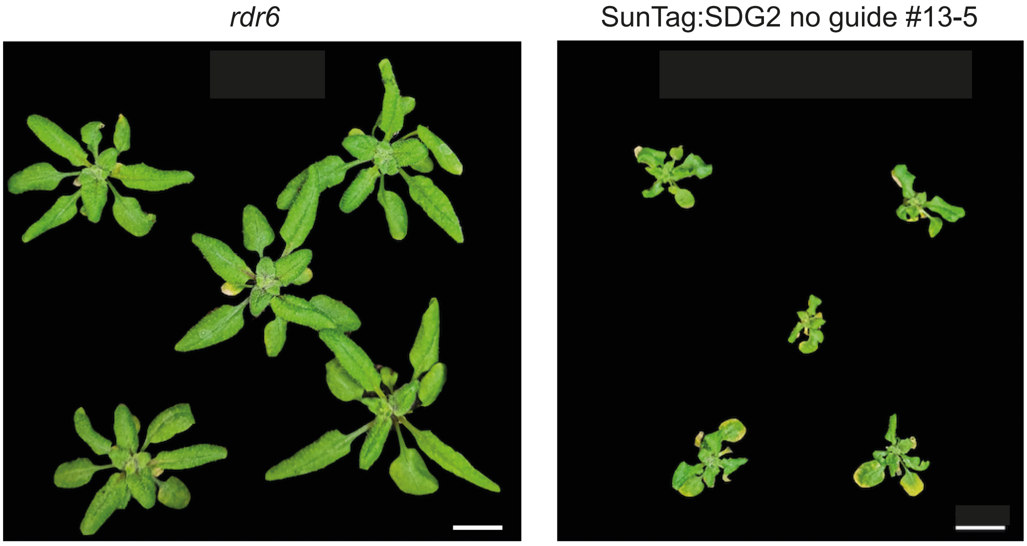

Supplementary Figure 14: Dwarfing phenotype of SunTag:SDG2 no guide lines as compared to *rdr6* (untransformed background) controls, representative images from 3-week old plants. Scale bar indicates 1cm.

S15

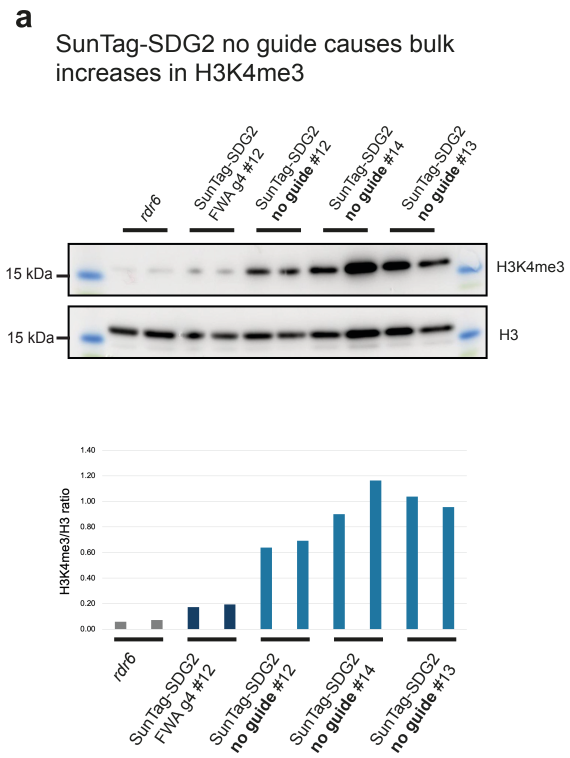

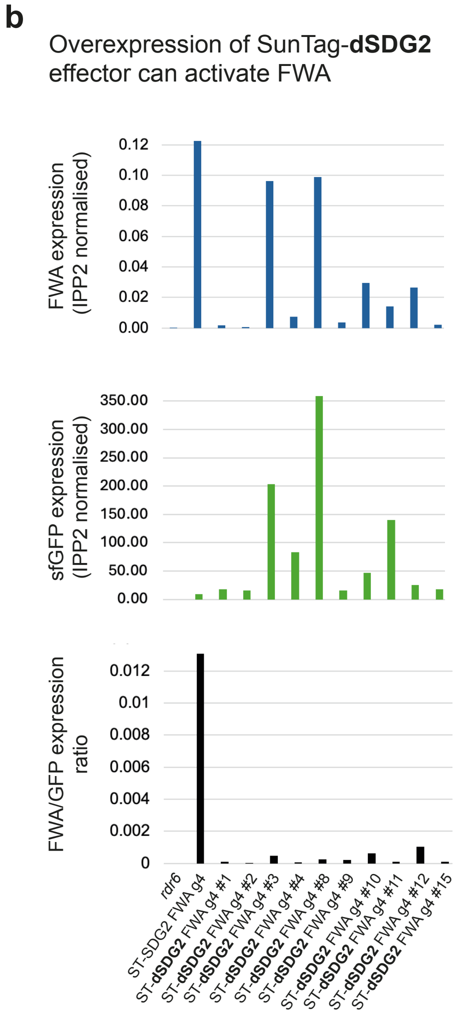

Supplementary Figure 15: A) Upper panel, bulk histone western blot showing levels of H3K4me3 in the genotypes indicated, H3 was used as a loading control. Lower panel, quantification of H3K4me3 levels in the genotypes indicated shown as H3K4me3/H3 ratio. B) RT-qPCR for expression of *FWA* (upper) and *sfGFP* (middle) and ratio (lower) in the genotypes indicated. Each bar is an individual line, sample is a pool of 10 seedlings.

S16

Genotype	T1's screened (no.)	FWA expressing (no.)	FWA expressing (%)
SunTag:SDG2:FWA_g4	23	19	83%
SunTag:dSDG2:FWA_g4	16	1	6%
SunTag:SDG2:No_g	14	2	14%

Supplementary Figure 16: Summary table showing the number of *FWA* activating T_1 lines per SunTag-SDG2 construct.

S17

a

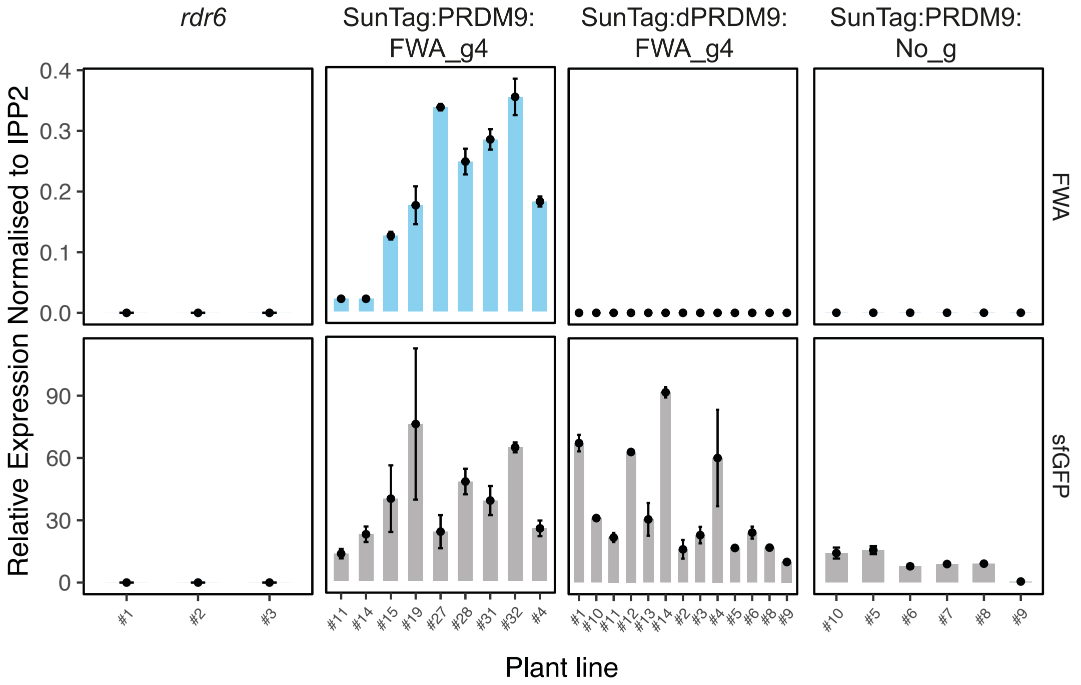

b

Genotype	T1's screened (no.)	FWA expressing (no.)	FWA expressing (%)
SunTag:PRDM9:FWA_g4	32	23	72%
SunTag:dPRDM9:FWA_g4	19	0	0%
SunTag:PRDM9:No_g	16	0	0%

Supplementary Figure 17: A) RT-qPCR of *FWA* expression (upper) and *sfGFP* expression (lower) for individual T_1 plants from the genotypes indicated. B) Summary table showing the number of FWA activating T_1 lines per SunTag-PRDM9 construct.

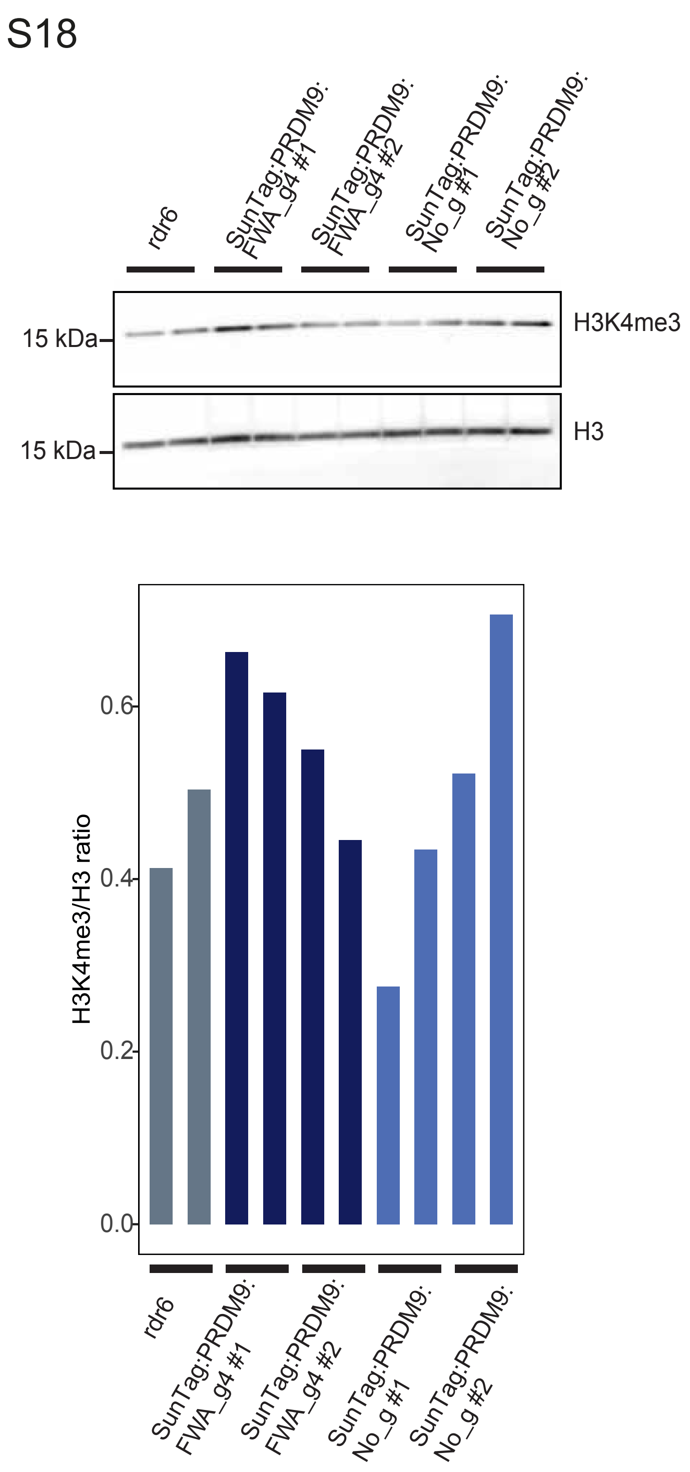

Supplementary Figure 18: Upper panel, bulk histone western blot showing levels of H3K4me3 in the genotypes indicated, H3 was used as a loading control. Lower panel, quantification of H3K4me3 levels in the genotypes indicated shown as H3K4me3/H3 ratio.

S19

a

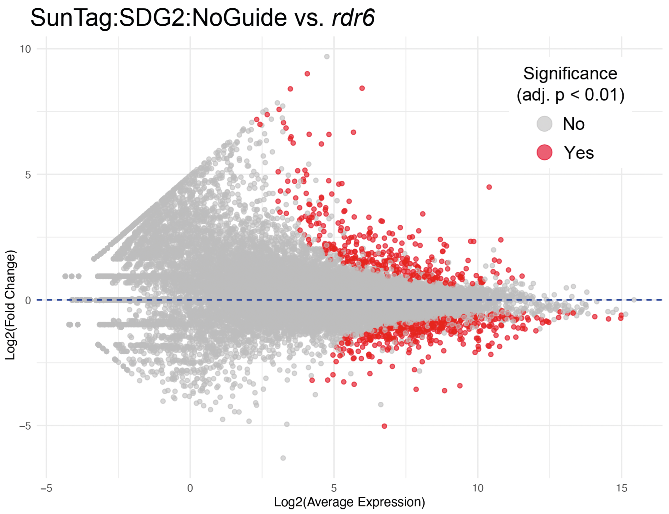

b

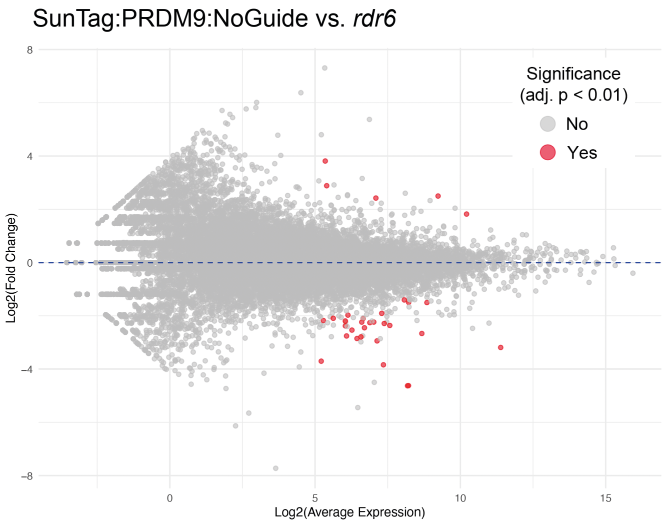

*Note that FWA is not a differentially expressed gene in either comparisons

Supplementary Figure 19: A) MA plot for transcriptome of SunTag:SDG2:No_g versus the non-transformed control (*rdr6*). B) MA plot for transcriptome of SunTag:PDRM9:No_g versus the non-transformed control (*rdr6*). Differentially expressed genes are depicted in red (FDR < 0.1). Note that *FWA* is not identified as differentially expressed in either comparison.

S20

a

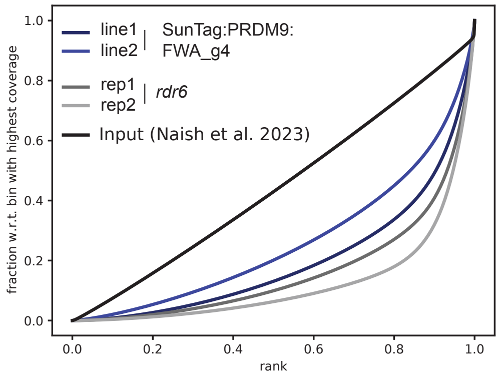

b

Sample	Total mapped reads	mapped reads in peaks	FrIP (%)
SunTag:PRDM9:FWA_g4 line1	38519016	23382610	60.7
SunTag:PRDM9:FWA_g4 line2	37332822	19114342	51.2
rdr6 rep1	23207046	14856926	64.0
rdr6 rep2	39630891	29971731	75.6

Supplementary Figure 20: Quality control for H3K4me3 ChIPseq of FWA targeting SunTag:PRDM9 lines. A) Fingerprint plot (DeepTools) B) Table of total mapped reads and fragments of reads in peaks (FRiP) scores.

S21

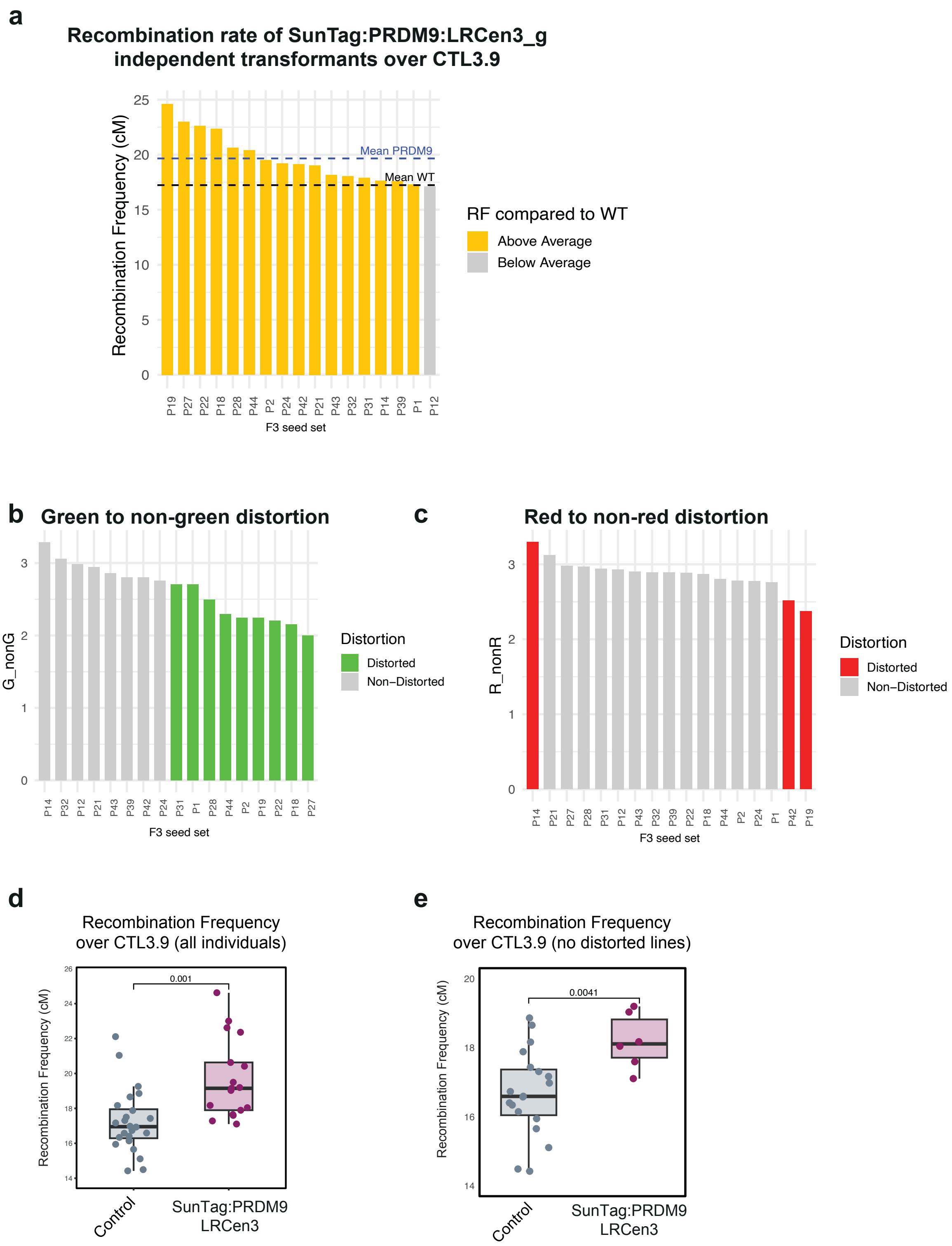
a
Recombination rate of SunTag:PRDM9:LRCen3_g
independent transformants over CTL3.9
Recombination Frequency (cM)
Mean PRDM9
Mean WT
F3 seed set
P19 P27 P22 P18 P28 P44 P2 P24 P42 P21 P43 P32 P31 P14 P39 P1 P12
RF compared to WT
Above Average
Below Average
b
Green to non-green distortion
G_nonG
F3 seed set
P14 P32 P12 P21 P43 P39 P42 P24 P31 P1 P28 P44 P2 P19 P22 P18 P27
Distortion
Distorted
Non−Distorted
c
Red to non-red distortion
R_nonR
F3 seed set
P14 P21 P27 P28 P31 P12 P43 P32 P39 P22 P18 P44 P2 P24 P1 P42 P19
Distortion
Distorted
Non−Distorted
d
Recombination Frequency
over CTL3.9 (all individuals)
Recombination Frequency (cM)
0.001
Control
SunTag:PRDM9
LRCen3
e
Recombination Frequency
over CTL3.9 (no distorted lines)
Recombination Frequency (cM)
0.0041
Control
SunTag:PRDM9
LRCen3

Supplementary Figure 21: A) Recombination rate over *CTL3.9* of the F_3 lines (as shown in main Figure 4). B) Distortion ratios for the green to non-green and C) red to non-red T-DNAs from *CTL3.9* from the F_3's. D) Recombination rate over *CTL3.9* (F_3's) including all scorable individuals. n = 24 for control, n = 17 for Suntag:PRDM9:LRCen3. E) Recombination rate over *CTL3.9* (F_3's) filtered for individuals with no distortion (i.e. 3:1). n = 19 for control, n = 6 for Suntag:PRDM9:LRCen3. *p*-values indicates two-sample students *t*-test.

Supplementary Table 1: Oligo List.

**SunTag cloning**	
SDG2_BsiWI_Fw	GAGGTCGGACCGGTCGTACG
SDG2_mutation_Rev	ATTAAAATCGAAAGTTATCTCCTCGCC
SDG2_mutation_Fw	GATTTTAATTCTGTAACTGAGAGTAAG
SDG2_BsiWI_Rev	CGAGATCCTCCTCCCGTACG
Universal F1	GTCATCTATGTTACGAAGCTTTCGTTGAACAACGG
Universal R2	TCGTGCTCCACCATGGTACCAAAAAAAGCACCGACTCGGTGC
pFWA_gRNA4_F2	acggaaagatgtatgggcttGTTTTAGAGCTAGAAATAGC
pFWA_gRNA4_R1	aagcccatacatctttccgtCAATCACTACTTCGACTCTA
pSNC1_gRNA4_F2	ctatgcaagtcttctaagaaGTTTTAGAGCTAGAAATAGC
pSNC1_gRNA4_R1	ttcttagaagacttgcatagCAATCACTACTTCGACTCTA
pSNC1_gRNA5_F2	atcaaatccctaacaagataGTTTTAGAGCTAGAAATAGC
pSNC1_gRNA5_R1	tatcttgttagggatttgatCAATCACTACTTCGACTCTA
LRCen3_g_F2	aggcttacaagattgggttg**GTTTTAGAGCTAGAAATAGC**
LRCen3_g_R1	caacccaatcttgtaagcct**CAATCACTACTTCGACTCTA**
**PRDM9 cloning into SunTag**	
RP_PRDM9_G282A_3'	cgagatcctcctcccgtacgtcaaa
FP_PRDM9_G282A_5'	GAGGTCGGACCGGTCGTACGTATC
RP_PRDM9_G282A_middle	CTCTGTGATCTGAGCCTCATAGGGGCCA
FP_PRDM9_G282A_middle	TGGCCCCTATGAGGCTCAGATCACAGAG
**ChIP-qPCRs**	
High_H3K4me3_AT5G65130_F	CCAGACATTGTTCGTCAAGGACAC
High_H3K4me3_AT5G65130_R	CAACGTTATATCCGACTCAGGAGATG
Low_H3K4me3_AT2G40004up_F	CAGCTACAAGTTCAACTAATCATGC
Low_H3K4me3_AT2G40004up_R	CTTTAATTGCCCGTCATTACGCTAC
pFWA_ChIP_Fw	AATATCAGATCTTGCGCCGCTC
pFWA_ChIP_Rev	GAAAAACAGACAAATCGGGAACC
pSNC1_ChIP_Fw1	ATGGCCTTATCTTGTTAGG
pSNC1_ChIP_Fw2	GAGTAGAAGCTTTCCAAGG
**RT-qPCRs**	
sfGFP_qPCR_Fw	CAAAGATGACGGGACCTACAA

sfGFP_qPCR_Rev	GTACTCGAGTTTGTGTCCAAGA
FWA_qPCR_Fw	TTAGATCCAAAGGAGTATCAAAG
FWA_qPCR_Rev	CTTTGGTACCAGCGGAGA
SNC1_qPCR_Fw	TTTGAAGAGACATGCAAGGC
SNC1_qPCR_Rev	TCGGCAAGCTCTTCAATCATGG
