# Supplementary material for: CRISPR targeting of H3K4me3 activates gene expression and unlocks centromere-proximal crossover recombination in Arabidopsis
Source: Nat Commun. 2025 Oct 31;16:9587. doi: 10.1038/s41467-025-65167-3 (PMC12578922; doi:10.1038/s41467-025-65167-3)
Supplement: Supplementary file 2 — Reporting Summary [file 41467_2025_65167_MOESM2_ESM.pdf]

nature portfolio

Corresponding author(s): C. Jake Harris

Last updated by author(s): Aug 1, 2025

Reporting Summary

Nature Portfolio wishes to improve the reproducibility of the work that we publish. This form provides structure for consistency and transparency in reporting. For further information on Nature Portfolio policies, see our Editorial Policies and the Editorial Policy Checklist.

Statistics

For all statistical analyses, confirm that the following items are present in the figure legend, table legend, main text, or Methods section.

n/a	Confirmed	
☐	☒	The exact sample size (*n*) for each experimental group/condition, given as a discrete number and unit of measurement
☐	☒	A statement on whether measurements were taken from distinct samples or whether the same sample was measured repeatedly
☐	☒	The statistical test(s) used AND whether they are one- or two-sided<br>*Only common tests should be described solely by name; describe more complex techniques in the Methods section.*
☒	☐	A description of all covariates tested
☐	☒	A description of any assumptions or corrections, such as tests of normality and adjustment for multiple comparisons
☐	☒	A full description of the statistical parameters including central tendency (e.g. means) or other basic estimates (e.g. regression coefficient) AND variation (e.g. standard deviation) or associated estimates of uncertainty (e.g. confidence intervals)
☐	☒	For null hypothesis testing, the test statistic (e.g. *F*, *t*, *r*) with confidence intervals, effect sizes, degrees of freedom and *P* value noted<br>*Give P values as exact values whenever suitable.*
☒	☐	For Bayesian analysis, information on the choice of priors and Markov chain Monte Carlo settings
☒	☐	For hierarchical and complex designs, identification of the appropriate level for tests and full reporting of outcomes
☒	☐	Estimates of effect sizes (e.g. Cohen's *d*, Pearson's *r*), indicating how they were calculated

Our web collection on statistics for biologists contains articles on many of the points above.

Software and code

Policy information about availability of computer code

Data collection	No software was used for data collection.
Data analysis	ChIP-seq analysis:<br>Bowtie2 (version 2.5.0) was used to map the PE150 bp read data in fastq format to the TAIR10 genome (--no-unal), and were converted to bam format using Samtools (version 1.10). Reads were de-duplicated using the samtools fixmate and markdup commands (see https://github.com/C-Jake-Harris/Binenbaum-SunTag-H3K4me3 for more details). Tracks were generated in DeepTools (version 3.5.1) using bamCoverage (--normalizeUsing RPGC, --effectiveGenomeSize 135000000 --binSize 10) with multicopy regions blacklisted (--blackListFileName) using the regions identified in (Klasfeld et al., 2022). For analysis of reads mapping to the centromere, a modified version of the previously described pipeline (Wlodzimierz et al., 2023) was used. Briefly, PE150 bp read data in fastq format were mapped to the Col-CEN genome (Naish et al., 2021) using Bowtie2 (version 2.2.5) with (--very-sensitive -k 200 --no-unal --no-discordant) and were filtered for primary alignments using samtools view (-F 256 -q 5) prior to deduplication and track generation, as above. Peaks were called using MACS2 (version 2.2.9.1) with default parameters. multiBigwigSummary (BED-file --outRawCounts) was used to gather normalised read enrichment over called peaks. Correlation plots were generated in DeepTools using MultiBamSummary (--binSize 25) and PlotCorrelation (-c pearson --removeOutliers --plotNumbers -p heatmap).<br><br>gRNA binding analysis:<br>For the LRCen3 binding and mismatch analysis, the LRCen3 guide RNA sequence (5'-AGGCTTACAAGATTGGGTTG-3'), was mapped to the Col-CEN genome using bowtie2 (version 2.2.5). The following options were used for no mismatches (-f -a --end-to-end --np 0 --score-min L,0,0) and for 1 mismatch (-f -a --end-to-end --score-min L,0,-1). Mapped reads were sorted and converted to bam format using Samtools (version 1.10), and subsequently converted to .bed format using the bamtobed function in bedtools (2.20.1). Bed files were used for downstream analysis in R to generate chromosome-wide density plots over 100kb regions.

For manuscripts utilizing custom algorithms or software that are central to the research but not yet described in published literature, software must be made available to editors and reviewers. We strongly encourage code deposition in a community repository (e.g. GitHub). See the Nature Portfolio guidelines for submitting code & software for further information.

Data

Policy information about availability of data

All manuscripts must include a data availability statement. This statement should provide the following information, where applicable:

- Accession codes, unique identifiers, or web links for publicly available datasets
- A description of any restrictions on data availability
- For clinical datasets or third party data, please ensure that the statement adheres to our policy

The high-throughput sequencing data generated has been deposited to NCBI (GSE288686). Previously published datasets: CENH3 (Wlodzimierz et al., 2023). Custom scripts used are available on GitHub (https://github.com/C-Jake-Harris/Binenbaum-SunTag-H3K4me3). Source Data are provided with this paper.

Research involving human participants, their data, or biological material

Policy information about studies with human participants or human data. See also policy information about sex, gender (identity/presentation), and sexual orientation and race, ethnicity and racism.

Reporting on sex and gender	N/A
Reporting on race, ethnicity, or other socially relevant groupings	N/A
Population characteristics	N/A
Recruitment	N/A
Ethics oversight	*Identify the organization(s) that approved the study protocol.*

Note that full information on the approval of the study protocol must also be provided in the manuscript.

Field-specific reporting

Please select the one below that is the best fit for your research. If you are not sure, read the appropriate sections before making your selection.

[x] Life sciences [ ] Behavioural & social sciences [ ] Ecological, evolutionary & environmental sciences

For a reference copy of the document with all sections, see nature.com/documents/nr-reporting-summary-flat.pdf

Life sciences study design

All studies must disclose on these points even when the disclosure is negative.

Sample size	Sample sizes are provided for each experiment. In general, we used the highest number of biological replicates feasible, considering material availability and technical constraints. For ChIP-seq (n = 2) and QuantSeq (n = 3), sample sizes follow ENCODE consortium guidelines. For other assays, sample sizes were chosen to ensure reproducibility and support robust statistical analysis.
Data exclusions	No data was excluded.
Replication	All findings were succefully reproduced across several replicates. Replicate numbers are specified in the figure legends and methods. For all gene expression assays 2 technical replicates were implemented for each biological replicate, to ensure consistency. No major inconsistencies were observed between replicates, and all data shown are representative of reproducible findings.
Randomization	For Pst::LUX infection assays the position of each line was randomized in the 96 well plates to minimize position effects. For the rest of the experiments each genotype was grown side by side with the control samples, with positions of pots in trays or of the plates being randomized where applicable.
Blinding	Due to the nature of the experimental setup, blinding was not applicable. All data was collected based on the genotype of the plants.

Reporting for specific materials, systems and methods

We require information from authors about some types of materials, experimental systems and methods used in many studies. Here, indicate whether each material, system or method listed is relevant to your study. If you are not sure if a list item applies to your research, read the appropriate section before selecting a response.

Materials & experimental systems

n/a	Involved in the study
☐	☒ Antibodies
☒	☐ Eukaryotic cell lines
☒	☐ Palaeontology and archaeology
☒	☐ Animals and other organisms
☒	☐ Clinical data
☒	☐ Dual use research of concern
☐	☒ Plants

Methods

n/a	Involved in the study
☐	☒ ChIP-seq
☐	☐ Flow cytometry
☐	☐ MRI-based neuroimaging

Antibodies

Antibodies used	anti-H3 (ab1791, Abcam), anti-H3K4me3 (ab8580, Abcam), anti-HA (3F10, Merck).
Validation	anti-H3 (ab1791, Abcam) - Validated by the manufacturer, https://www.abcam.com/en-us/products/primary-antibodies/histone-h3-antibody-nuclear-marker-and-chip-grade-ab1791#tab=datasheet<br>anti-H3K4me3 (ab8580, Abcam) - Validated by the manufacturer, https://www.abcam.com/en-us/products/primary-antibodies/histone-h3-tri-methyl-k4-antibody-chip-grade-ab8580?srsltid=AfmBOoosgyBHJAgDdYq9WaxephxrblWMwXz7cLpHq4TPHSXtHyH4jzUh<br>anti-HA (3F10, Merck) - Was validated by manufacturer, https://www.sigmaaldrich.com/GB/en/product/roche/12158167001#product-documentation

Dual use research of concern

Policy information about dual use research of concern

Hazards

Could the accidental, deliberate or reckless misuse of agents or technologies generated in the work, or the application of information presented in the manuscript, pose a threat to:

No	Yes
☒	☐ Public health
☒	☐ National security
☒	☐ Crops and/or livestock
☒	☐ Ecosystems
☒	☐ Any other significant area

Experiments of concern

Does the work involve any of these experiments of concern:

No	Yes
☒	☐ Demonstrate how to render a vaccine ineffective
☒	☐ Confer resistance to therapeutically useful antibiotics or antiviral agents
☒	☐ Enhance the virulence of a pathogen or render a nonpathogen virulent
☒	☐ Increase transmissibility of a pathogen
☒	☐ Alter the host range of a pathogen
☒	☐ Enable evasion of diagnostic/detection modalities
☒	☐ Enable the weaponization of a biological agent or toxin
☒	☐ Any other potentially harmful combination of experiments and agents

Plants

Seed stocks: rdr6-15, fwa rdr6-15, Col-0, edr1-1, NahG, bal

Novel plant genotypes: SunTag-SDG2 targeting FWA in rdr6-15 background, SunTag-SDG2 targeting SNC1 in rdr6-15 background, SunTag-SDG2 targeting the centromeric CEN178 repeat in CTL3.9 background, SunTag-PRDM9 targeting FWA in rdr6-15 background and SunTag-PRDM9 targeting the centromeric CEN178 repeat in CTL3.9 background. All these novel plant genotypes were generated using agrobacterium-mediated transformation.

Authentication: The seed stocks were genotyped and are frequently used in multiple previous studies. The novel transgenic lines were grown on a selective media and checked for GFP expression and GFP fluorescence.

ChIP-seq

Data deposition

☒ Confirm that both raw and final processed data have been deposited in a public database such as GEO.

☒ Confirm that you have deposited or provided access to graph files (e.g. BED files) for the called peaks.

Data access links
May remain private before publication.

The data has been deposited to GEO under the accession (GSE288686) and is available with the reviewer access token (ybobyyqozfabtmt).

Files in database submission

GSM8773045 ChIPseq_SetB_antiHA_rdr6_rep1
GSM8773046 ChIPseq_SetB_antiHA_rdr6_rep2
GSM8773047 ChIPseq_SetB_antiHA_StSDG2g4_lineCB10_rep1
GSM8773048 ChIPseq_SetB_antiHA_StSDG2g4_lineCB10_rep2
GSM8773049 ChIPseq_SetB_antiHA_StSDG2g4_lineCB12_rep1
GSM8773050 ChIPseq_SetB_antiHA_StSDG2g4_lineCB12_rep2
GSM8773051 ChIPseq_SetC_antiH3K4me3_Col0_rep1
GSM8773052 ChIPseq_SetC_antiH3K4me3_Col0_rep2
GSM8773053 ChIPseq_SetC_antiH3K4me3_StSDG2gLRCEN3_noST_rep1
GSM8773054 ChIPseq_SetC_antiH3K4me3_StSDG2gLRCEN3_noST_rep2
GSM8773055 ChIPseq_SetC_antiH3K4me3_StSDG2gLRCEN3_withST_rep1
GSM8773056 ChIPseq_SetC_antiH3K4me3_StSDG2gLRCEN3_withST_rep2
GSM8773057 ChIPseq_SetC_antiHA_Col0_rep1
GSM8773058 ChIPseq_SetC_antiHA_Col0_rep2
GSM8773059 ChIPseq_SetC_antiHA_StSDG2gLRCEN3_noST_rep1
GSM8773060 ChIPseq_SetC_antiHA_StSDG2gLRCEN3_noST_rep2
GSM8773061 ChIPseq_SetC_antiHA_StSDG2gLRCEN3_withST_rep1
GSM8773062 ChIPseq_SetC_antiHA_StSDG2gLRCEN3_withST_rep2
GSM8773063 ChIPseq_SetD_antiH3K4me3_rdr6_rep1
GSM8773064 ChIPseq_SetD_antiH3K4me3_rdr6_rep2
GSM8773065 ChIPseq_SetD_antiH3K4me3_StPRDM9g4_line1
GSM8773066 ChIPseq_SetD_antiH3K4me3_StPRDM9g4_line2
GSM8773067 ChIPseq_SetD_antiHA_rdr6_rep1
GSM8773068 ChIPseq_SetD_antiHA_rdr6_rep2
GSM8773069 ChIPseq_SetD_antiHA_StPRDM9g4_line1
GSM8773070 ChIPseq_SetD_antiHA_StPRDM9g4_line2
GSM8773071 QuantSeq_SetA_rdr6_rep1
GSM8773072 QuantSeq_SetA_rdr6_rep2
GSM8773073 QuantSeq_SetA_rdr6_rep3
GSM8773074 QuantSeq_SetA_StPRDM9g4_rep1
GSM8773075 QuantSeq_SetA_StPRDM9g4_rep2
GSM8773076 QuantSeq_SetA_StPRDM9g4_rep3
GSM8773077 QuantSeq_SetA_StPRDM9noG_rep1
GSM8773078 QuantSeq_SetA_StPRDM9noG_rep2
GSM8773079 QuantSeq_SetA_StPRDM9noG_rep3
GSM8773080 QuantSeq_SetE_rdr6_rep1
GSM8773081 QuantSeq_SetE_rdr6_rep2
GSM8773082 QuantSeq_SetE_rdr6_rep3
GSM8773083 QuantSeq_SetE_StSDG2g4_rep1
GSM8773084 QuantSeq_SetE_StSDG2g4_rep2
GSM8773085 QuantSeq_SetE_StSDG2g4_rep3
GSM8773086 QuantSeq_SetE_StSDG2noG_rep1
GSM8773087 QuantSeq_SetE_StSDG2noG_rep2

GSM8773088 QuantSeq_SetE_StSDG2noG_rep3
GSM9144061 ChIPseq_SetB_antiH3K4me3_rdr6_rep1
GSM9144062 ChIPseq_SetB_antiH3K4me3_rdr6_rep2
GSM9144063 ChIPseq_SetB_antiH3K4me3_StSDG2g4_lineCB10_rep1
GSM9144064 ChIPseq_SetB_antiH3K4me3_StSDG2g4_lineCB10_rep2
GSM9144065 ChIPseq_SetB_antiH3K4me3_StSDG2g4_lineCB12_rep1
GSM9144066 ChIPseq_SetB_antiH3K4me3_StSDG2g4_lineCB12_rep2

Genome browser session (e.g. UCSC): NA

Methodology

Replicates: 2 for ChIP-seq, 3 for QuantSeq

Sequencing depth: ChIP-seq libraies were sequenced at a depth of >3Gb, QuantSeq samples were sequenced at 1Gb depth.

Antibodies: anti-H3 (ab1791, Abcam), anti-H3K4me3 (ab8580, Abcam), anti-HA (3F10, Merck).

Peak calling parameters: Default parametars

Data quality: Standard QC metrics were used to assess the libraries, including fingerprint plots, and FrIP scores and MACS peak calling.

Software:
Bowtie2 (version 2.5.0)
Samtools (version 1.10)
MACS2 (version 2.2.9.1)
DeepTools (version 3.5.1)
bedtools (2.20.1)
cutadapt (version 1.18)
Star (version 2.7.10b)
R Studio (Version 2024.12.1+563)

Flow Cytometry

Plots

Confirm that:

- [ ] The axis labels state the marker and fluorochrome used (e.g. CD4-FITC).
- [ ] The axis scales are clearly visible. Include numbers along axes only for bottom left plot of group (a 'group' is an analysis of identical markers).
- [ ] All plots are contour plots with outliers or pseudocolor plots.
- [ ] A numerical value for number of cells or percentage (with statistics) is provided.

Methodology

Sample preparation: *Describe the sample preparation, detailing the biological source of the cells and any tissue processing steps used.*

Instrument: *Identify the instrument used for data collection, specifying make and model number.*

Software: *Describe the software used to collect and analyze the flow cytometry data. For custom code that has been deposited into a community repository, provide accession details.*

Cell population abundance: *Describe the abundance of the relevant cell populations within post-sort fractions, providing details on the purity of the samples and how it was determined.*

Gating strategy: *Describe the gating strategy used for all relevant experiments, specifying the preliminary FSC/SSC gates of the starting cell population, indicating where boundaries between "positive" and "negative" staining cell populations are defined.*

- [ ] Tick this box to confirm that a figure exemplifying the gating strategy is provided in the Supplementary Information.

Magnetic resonance imaging

Experimental design

Design type: *Indicate task or resting state; event-related or block design.*

Design specifications	*Specify the number of blocks, trials or experimental units per session and/or subject, and specify the length of each trial or block (if trials are blocked) and interval between trials.*
Behavioral performance measures	*State number and/or type of variables recorded (e.g. correct button press, response time) and what statistics were used to establish that the subjects were performing the task as expected (e.g. mean, range, and/or standard deviation across subjects).*

Acquisition

Imaging type(s)	*Specify: functional, structural, diffusion, perfusion.*
Field strength	*Specify in Tesla*
Sequence & imaging parameters	*Specify the pulse sequence type (gradient echo, spin echo, etc.), imaging type (EPI, spiral, etc.), field of view, matrix size, slice thickness, orientation and TE/TR/flip angle.*
Area of acquisition	*State whether a whole brain scan was used OR define the area of acquisition, describing how the region was determined.*

Diffusion MRI ☐ Used ☐ Not used

Preprocessing

Preprocessing software	*Provide detail on software version and revision number and on specific parameters (model/functions, brain extraction, segmentation, smoothing kernel size, etc.).*
Normalization	*If data were normalized/standardized, describe the approach(es): specify linear or non-linear and define image types used for transformation OR indicate that data were not normalized and explain rationale for lack of normalization.*
Normalization template	*Describe the template used for normalization/transformation, specifying subject space or group standardized space (e.g. original Talairach, MNI305, ICBM152) OR indicate that the data were not normalized.*
Noise and artifact removal	*Describe your procedure(s) for artifact and structured noise removal, specifying motion parameters, tissue signals and physiological signals (heart rate, respiration).*
Volume censoring	*Define your software and/or method and criteria for volume censoring, and state the extent of such censoring.*

Statistical modeling & inference

Model type and settings	*Specify type (mass univariate, multivariate, RSA, predictive, etc.) and describe essential details of the model at the first and second levels (e.g. fixed, random or mixed effects; drift or auto-correlation).*
Effect(s) tested	*Define precise effect in terms of the task or stimulus conditions instead of psychological concepts and indicate whether ANOVA or factorial designs were used.*

Specify type of analysis: ☐ Whole brain ☐ ROI-based ☐ Both

Statistic type for inference (See Eklund et al. 2016)	*Specify voxel-wise or cluster-wise and report all relevant parameters for cluster-wise methods.*
Correction	*Describe the type of correction and how it is obtained for multiple comparisons (e.g. FWE, FDR, permutation or Monte Carlo).*

Models & analysis

n/a	Involved in the study
☐	☐ Functional and/or effective connectivity
☐	☐ Graph analysis
☐	☐ Multivariate modeling or predictive analysis

Functional and/or effective connectivity	*Report the measures of dependence used and the model details (e.g. Pearson correlation, partial correlation, mutual information).*
Graph analysis	*Report the dependent variable and connectivity measure, specifying weighted graph or binarized graph, subject- or group-level, and the global and/or node summaries used (e.g. clustering coefficient, efficiency, etc.).*
Multivariate modeling and predictive analysis	*Specify independent variables, features extraction and dimension reduction, model, training and evaluation metrics.*
